# The Impact of Gut Microbial Metabolomics on Type 2 Diabetes Development in People Living with HIV

**DOI:** 10.3390/metabo15090627

**Published:** 2025-09-19

**Authors:** Yusnier Lázaro Díaz-Rodríguez, Elsa Janneth Anaya-Ambriz, Paula Catalina Méndez-Ríos, Jaime F. Andrade-Villanueva, Luz A. González-Hernández, Tania Elisa Holguín-Aguirre, Pedro Martínez-Ayala, Vida V. Ruiz-Herrera, Monserrat Alvarez-Zavala, Karina Sánchez-Reyes

**Affiliations:** 1 Programa de Maestría en Microbiología Médica, Centro Universitario de Ciencias de la Salud, Universidad de Guadalajara, Guadalajara 44340, Mexico; yusnier.diaz9426@alumnos.udg.mx; 2 Programa de Doctorado en Microbiología Médica, Centro Universitario de Ciencias de la Salud, Universidad de Guadalajara, Guadalajara 44340, Mexico; elsa.anaya@academicos.udg.mx; 3 Programa de Doctorado en Ciencias en Biología Molecular en Medicina, Centro Universitario de Ciencias de la Salud, Universidad de Guadalajara, Guadalajara 44340, Mexico; 4 Unidad de VIH, Hospital Civil de Guadalajara “Fray Antonio Alcalde”, Guadalajara 44350, Mexico; drjandradev@gmail.com (J.F.A.-V.); luceroga08@gmail.com (L.A.G.-H.); taniaholguinaguirre@gmail.com (T.E.H.-A.); pedro.martinez@cucs.udg.mx (P.M.-A.); vida.ruiz@academicos.udg.mx (V.V.R.-H.); montserrat.zavala@academicos.udg.mx (M.A.-Z.); 5 Departamento de Clínicas Médicas, Instituto de Investigación en Inmunodeficiencias y VIH (InIVIH), Centro Universitario de Ciencias de la Salud, Universidad de Guadalajara, Guadalajara 44350, Mexico

**Keywords:** type 2 diabetes, dysbiosis, HIV infection, metabolomics, microbiota, microbial metabolites

## Abstract

**Background/Objectives:** HIV infection has been associated with an increased incidence of non-communicable comorbidities, including metabolic disorders. This phenomenon has been linked to gut microbiota dysbiosis, which involves not only changes in bacterial composition but also functional alterations in metabolite production. The objective of this study was to describe the impact of intestinal microbial metabolomics on the development of type 2 diabetes in people living with HIV. **Methods:** This study provides a narrative synthesis of current evidence addressing the role of gut microbiota-derived metabolites in immunometabolic regulation and their implications in HIV-associated type 2 diabetes. **Results:** Microbial metabolites play a fundamental role in regulating key physiological processes such as intestinal permeability, systemic immune activation, and glucose metabolism. Compounds such as short-chain fatty acids, tryptophan catabolites, secondary bile acids, trimethylamine N-oxide, and imidazole propionate have been shown to significantly influence immunometabolic balance. In people living with HIV, these microbial products may exert diverse effects depending on their chemical nature and the molecular pathways they activate in peripheral tissues. The interaction between dysbiosis, chronic low-grade inflammation, and HIV-associated metabolic disturbances may contribute to the early onset of type 2 diabetes beyond traditional risk factors. **Conclusions:** Recognizing the role of microbial metabolites in the context of HIV infection is essential to broaden our pathophysiological understanding of associated metabolic comorbidities. It also opens opportunities to develop more comprehensive diagnostic and therapeutic strategies that include modulation of the gut microbiota and its metabolic activity for the prevention and management of type 2 diabetes in this population.

## 1. Introduction

By the end of 2023, approximately 39.9 million people were living with the human immunodeficiency virus (HIV) worldwide. Of these, 1.3 million corresponded to new diagnoses, and 630,000 died from acquired immunodeficiency syndrome (AIDS)-related illnesses. Of the total number of people living with HIV (PLWHIV), 89% were receiving antiretroviral therapy (ART) [1]. In Mexico, as of November 2024, 15,798 new cases had been registered, accumulating a total of 167,947 diagnosed cases since 2014 [2].

The progression of the disease in PLWHIV has been consistently associated with an increased risk of developing non-communicable chronic diseases, especially type 2 diabetes (T2D). This risk can be up to four times higher, in addition to occurring at younger ages and not being related to obesity. This phenomenon has been linked in the scientific literature to alterations in the gut microbiota (GM), which are associated with increased microbial translocation, low-grade systemic inflammation, and changes in the metabolite profile derived from said dysbiosis. The presence and concentration of these metabolites in the blood vary in PLWHIV, as they participate in diverse pathophysiological processes, depending on their chemical structure and the molecular mechanisms they activate [3,4].

The impact of metabolomics on the study of the GM has enabled the identification of key biomarkers and biochemical pathways related to HIV, associated intestinal dysbiosis, and alterations in glycemic homeostasis. However, the specific mechanisms and their clinical relevance remain unclear. For these reasons, the objective of this review is to describe the impact of intestinal microbial metabolomics on the development of T2D in PLWHIV.

## 2. Gut Microbiota Alterations in People Living with HIV

After contact via the sexual route of HIV-infected secretions with mucosal surfaces, the virus crosses through them. This phenomenon has major implications at the level of mucosa-associated lymphoid tissue (especially in the GALT), as it compromises immunological homeostasis and alters the microbiota, favoring the overgrowth of pathogenic species, microbial translocation into the bloodstream, and a characteristic chronic systemic inflammation of this infection [5]. In the alterations at the GALT level, the virus’s tropism for cells expressing CD4 and co-receptors plays a key role, these may include the chemokine receptor CCR5 in early infections, or the CXC motif chemokine receptor CXCR4 in chronic infections, which are expressed on dendritic cells, memory T lymphocytes, mucosal-associated T lymphocytes, and cells of the monocyte–macrophage lineage [6].

Thus, a massive depletion occurs of memory CD4^+^ T lymphocytes that express CCR5, due to their high prevalence in the GALT, particularly specialized subpopulations such as Th17 and Th22 cells. Up to 20% of these lymphocytes can become infected, and another 60% die by apoptosis after activation, resulting in a loss of up to 80% in the first three weeks of infection. Despite peripheral immune reconstitution, the loss of CD4^+^ T lymphocytes in the GALT is not recovered [7,8]. In the case of B lymphocytes, although they do not experience a significant numerical reduction in the initial phases of infection, their functionality is compromised due to damage to other cell populations essential for the formation and maintenance of germinal centers. It has been documented that up to 50% of these centers in the intestine may disappear within the first 80 days post-infection [9].

Th17 and Th22 cells perform essential functions in preserving intestinal mucosal integrity and regulating immune responses against commensal and pathogenic microorganisms. For example, the secretion of IL-22 by Th17 cells, mediated by their intrinsic glutathione production, is crucial for maintaining intestinal barrier integrity and gut microbiological homeostasis [10]. However, it has been described that HIV can also polarize CD4^+^ T lymphocytes toward the Th17 phenotype, thereby inhibiting antiviral elements and ensuring the persistence of the infection. Through the study of sequences of the virus’s long terminal repeat (LTR) regions, the presence of binding sites for RORγt, a transcription factor that determines the Th17 phenotype, has been observed, which explains the possibility that its activation may modulate provirus expression. In addition, in infected antigen-presenting cells, the HIV-1 Tat protein activates the STAT-3 factor, thereby increasing viral replication [11,12]. STAT-3 also interacts with the NF-κB pathway, thus amplifying the signal that stimulates HIV expression [13].

Secondary to these alterations in immunological homeostasis in the context of HIV infection, changes in microbiota composition have been documented with greater frequency. Among these changes is an increase in the phylum Pseudomonadota, particularly in genera such as *Prevotella* and members of the family Enterobacteriaceae, including potentially pathogenic species like *Escherichia-Shigella*, *Megasphaera*, *Klebsiella*, *Succinivibrio*, and *Ruminococcus gnavus*. Simultaneously, a reduction in beneficial genera such as *Bacteroides* and members of the Clostridia clade has been observed, which could compromise immunoregulatory functions and intestinal barrier integrity [14,15].

To the alterations caused by HIV infection itself in the microbiota are added other factors that determine the degree of dysbiosis: geographical location, sexual preferences, age, route of infection, and ART [14]. Regarding ART, several studies have shown that antiretroviral therapy may alter microbiota composition at different taxonomic levels. However, the mechanisms responsible for these changes are not fully understood, nor has it been precisely identified which bacteria are targeted by different drug types or how these modifications contribute to the development of dysbiosis. Among the hypotheses described are the direct antimicrobial effect of certain antiretrovirals, while others are based on pharmacokinetic characteristics such as distribution and absorption in the intestine, which may determine their concentration in different body compartments. It is also proposed that some of these agents could induce local inflammatory responses in the intestinal mucosa, causing increased epithelial permeability [15,16].

Altogether, GALT immune dysfunction and the consequent associated dysbiosis lead to disruption of the intestinal epithelial barrier, enterocyte apoptosis, and alteration of tight junctions, allowing microbial components to pass into the systemic circulation, a phenomenon known as microbial translocation. These components constitute the so-called pathogen-associated molecular patterns (PAMPs), which, once in circulation, are recognized by pattern recognition receptors (PRRs), triggering signaling cascades involving transcription factors such as NF-κB, with consequent production of inflammatory mediators such as TNF-α, IL-6, and IL-1β. This process persistently activates the systemic immune system, generating a chronic low-grade inflammatory state characteristic of HIV infection, even in people under effective ART [15,17].

In a study by Krug et al., it was shown that in PLWHIV without ART, intestinal permeability at the duodenal and colonic levels increases up to fourfold compared to seronegative individuals. This investigation was based on the evaluation of macromolecule permeability via measurements of FITC-Dextran 4000 flow and horseradish peroxidase. Likewise, it was observed that microbial translocation may occur via transcytosis or through filtration across apoptotic regions of the intestinal epithelium [18]. Another work by Tincati et al. included PLWHIV in primary and chronic infection, all ART-naive. They concluded that infection progression is accompanied by increased microbial translocation, supported by decreased expression of tight-junction proteins and increased systemic immune activation [19]. These findings are consistent with other investigations emphasizing the primary role of this persistent inflammatory response secondary to alterations in intestinal permeability in the pathophysiology of infection. Markers such as zonulin, intestinal fatty acid-binding protein (I-FABP), regenerating islet-derived protein 3α (REG3α), as well as lipopolysaccharide (LPS) and (1,3)-β-d-glucan (BDG), are indicators of intestinal damage and microbial translocation associated with systemic inflammation [20,21].

Overall, each of the alterations described, together with modifications in the composition and function of the microbial ecosystem, as well as variations in concentrations of different metabolites, elevated C-reactive protein and leptin, together with reduced adiponectin, promote a higher risk of metabolic comorbidities, particularly T2D, even in the absence of obesity [22]. Few studies have addressed the triad HIV–intestinal dysbiosis–T2D, particularly through evaluation of functional microbial metabolites. One such study was performed between 2015 and 2016 by Moon et al., who investigated African American and Hispanic women living with HIV on long-term ART, where they observed a relationship between bacterial translocation and chronic low-grade inflammatory state [23].

Likewise, the authors highlighted the importance of functional microbial metabolites such as tryptophan catabolism derivatives and branched-chain amino acids, as these elements are key in the link between dysbiosis and immunometabolic alteration. Additionally, an inverse correlation was reported between bacteria of the genera *Anaerococcus* and *Finegoldia* and inflammatory and metabolic stress markers, reinforcing the role of beneficial commensal bacteria with protective functions in immune activation and energy metabolism in PLWHIV and T2D [23]. Luo et al. expanded on these results in a larger cohort, in which they found elevated levels of microbial metabolites and biomarkers denoting an inflammatory environment in PLWHIV, especially with detectable viral load. Moreover, significant associations were reported between bacteria of the genera *Escherichia*, *Shigella*, and *Megasphaera* with T2D, while *Faecalibacterium* and *Adlercreutzia* showed inverse relationships. Thus, they consistently concluded that dysbiosis, inflammation, and particularly microbial metabolites play a key role in the link between HIV and T2D [24].

## 3. Metabolomic Alterations in HIV Infection and Type 2 Diabetes Associated with Intestinal Microbiota Dysbiosis

Microbial metabolites are compounds produced and/or directly or indirectly influenced at the intestinal level by the microorganisms present there, and which exert various systemic functions after entering the circulation. The production of these metabolites by the altered GM in PLWHIV and individuals with T2D constitutes the link between intestinal dysbiosis and such diseases [25] (Table 1).

### 3.1. Metabolites Derived from Tryptophan Catabolism

**Figure 1:**
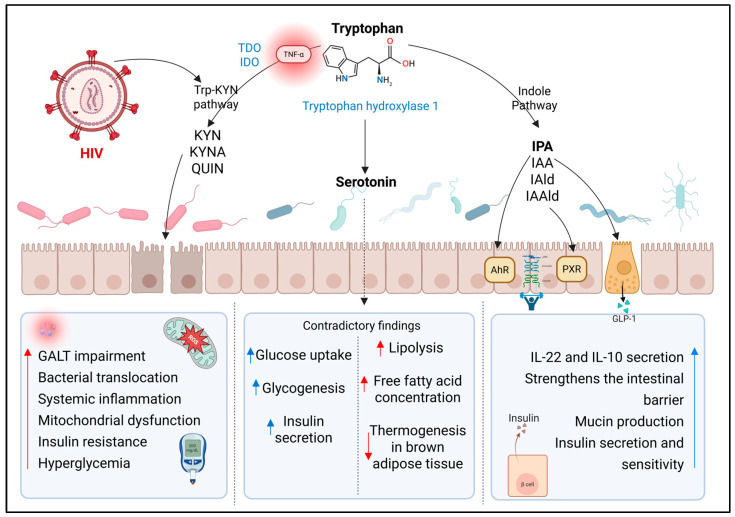
Tryptophan catabolism-derived metabolites and their impact on the development of T2D. Representation of metabolites derived from tryptophan catabolism, produced by the gut microbiota. The kynurenine, indole, and serotonin synthesis pathways are included. Aryl hydrocarbon receptor (AhR); glucagon-like peptide-1 (GLP-1); indole-3-acetaldehyde (IAAld); indole-3-acetic acid (IAA); indole-3-aldehyde (IAld); indole-3-propionic acid (IPA); indoleamine 2,3-dioxygenase (IDO); kynurenic acid (KYNA); kynurenine (KYN); kynurenine pathway (Trp-KYN); pregnane X receptor (PXR); quinolinic acid (QUIN); tryptophan 2,3-dioxygenase (TDO); tumor necrosis factor alpha (TNF-α). ↑ increase; ↓ decrease. Created with BioRender.com (accessed on 12 September 2025).

Tryptophan is an essential amino acid whose metabolism mainly follows three pathways: the serotonergic pathway, the kynurenine pathway (Trp-KYN), and the indole pathway, the latter mediated by the GM. These routes are modulated by various factors, such as inflammation, microbial composition, and the host’s immune status [25] (Figure 1).

#### 3.1.1. Kynurenine Pathway (Trp-KYN)

The Trp-KYN pathway generally develops in the presence of inflammation, constituting under these conditions the main destination of tryptophan (Trp). This pathway is regulated by two key enzymes: tryptophan 2,3-dioxygenase (TDO), mainly present in the liver, and indoleamine 2,3-dioxygenase (IDO), which exists in two isoforms (IDO1 and IDO2) and is expressed in immune and intestinal epithelial cells. In this way, several bioactive metabolites are generated, such as kynurenine (KYN), kynurenic acid (KYNA), anthranilic acid (AA), 3-hydroxykynurenine (3-HK), and quinolinic acid (QUIN), which have been implicated in immunomodulatory, neuroactive, and pro-oxidant functions [25,26,27] (Figure 1).

HIV infection chronically activates the Trp-KYN pathway, especially in the absence of ART, which is reflected in an elevated proportion of KYN relative to Trp. This activation is promoted by proinflammatory cytokines (TNF-α and IL-1β), which induce increased expression of IDO1, both in immune and intestinal epithelial cells [28,29,30,31]. The increased KYN/Trp ratio, marked by IDO activation, is associated with chronic inflammation, adipose tissue fibrosis, increased HOMA-IR, increased risk of T2D, and endothelial dysfunction. This is especially relevant in PLWHIV, where persistent immune activation and intestinal dysbiosis promote this metabolic shift, thus increasing the risk of T2D and cardiovascular [32,33,34]. It has been documented that this shift in Trp catabolism is related to disease progression, CD4^+^ T cell depletion, and systemic immune dysfunction. In addition, intestinal bacteria with high abundance in the microbiota of advanced-stage PLWHIV also express enzymes similar to IDO, promoting the conversion of Trp into kynurenines. Vujkovic-Cvijin et al. reported intestinal bacterial genera expressing genetic homologs to IDO1 in PLWHIV. Among those mentioned are *Pseudomonas*, *Xanthomonas*, *Burkholderia*, *Stenotrophomonas*, and *Shewanella* within the phylum Pseudomonadota, and members of the genus *Bacillus* within the phylum Bacillota [30].

In the case of the metabolites KYNA and AA, they have been related to progressive deterioration of mucosal immunity, favoring bacterial translocation and perpetuating inflammation. In addition, these compounds promote the imbalance between Th17 and Treg cells, aggravating immunosuppression and intestinal epithelial damage. QUIN and 3-HK, for their part, induce oxidative stress and mitochondrial dysfunction, affecting the immune response and exacerbating chronic inflammation [30].

The shift of Trp toward the kynurenine pathway has also been linked to the pathophysiology of T2D; that is, the overexpression of TDO/IDO induced by low-grade inflammation and intestinal dysbiosis reduces the availability of this amino acid for other, more beneficial catabolic pathways such as the production of indoles and serotonin [27]. QUIN and 3-HK, as explained previously, increase the levels of reactive oxygen species, alter the function of antioxidant enzymes, and contribute to IR [35]. A study in five multiethnic cohorts showed that elevated levels of KYN and QUIN are associated with a higher risk of developing T2D, and that diets rich in animal protein favor this metabolic profile [32].

#### 3.1.2. Indole Pathway

Another of the Trp catabolism pathways, mediated by the GM, is characterized by transforming this amino acid into a variety of indoles. Among these indoles are: indole-3-acetic acid (IAA), indole-3-aldehyde (IAld), indole-3-propionic acid (IPA), indoleacrylic acid, and indole-3-acetaldehyde (IAAld). These compounds interact with nuclear receptors such as the Aryl hydrocarbon Receptor (AhR) and the Pregnane X Receptor (PXR), thereby modulating immune functions, intestinal barrier integrity, and the production of cytokines such as IL-22 and IL-10 [25,36,37] (Figure 1).

In PLWHIV, especially in advanced stages of the disease, a reduction in indole production has been observed, which has been related to a decrease in IL-22 secretion by innate lymphocytes, alterations in intestinal epithelial barrier integrity, and overgrowth of pathogenic bacterial species [28,29,38]. This deficiency is also associated with lower AhR activation, reducing the expression of genes involved in intestinal barrier protection and exacerbating dysbiosis. Studies have identified that bacterial genera producing IPA, such as *Roseburia*, *Eubacterium*, *Lachnospira*, and *Coprobacter*, are decreased in PLWHIV, which could contribute to the development of cardiovascular and metabolic comorbidities [39]. On the other hand, in PLWHIV on HART, IAA has been associated with carotid intima-media thickness (c-IMT), and its progression event has not been observed in naive patients or in individuals negative for the infection [40]. In the case of indoleacrylic acid produced by *Parabacteroides distasonis*, it has been reported that increasing levels of IL-22 restore intestinal integrity and reduce metabolic inflammation through the activation of the AhR signaling pathway [41]. IAld also improves intestinal barrier, increasing goblet cells and markers such as Muc2 and reducing levels of IL-6, IL-1β, and TNF-α, through the inhibition of NF-kB [42,43].

IPA has been especially studied for its ability to protect against T2D. In a clinical trial it of 15-year follow-up, it was reported that patients that patients who developed diabetes had lower levels of IPA compared to people who did not develop T2D, and IPA levels were negatively correlated with hs-CRP [44,45]. This metabolite strengthens the intestinal barrier by stimulating the production of mucins, Trefoil Factor Family 3 (TFF3), resistin-like molecule beta (RELMβ), and immunoregulatory cytokines such as IL-10. Furthermore, it reduces TNF-α production in enterocytes through PXR activation, which improves intestinal homeostasis in inflammatory states [46]. IPA has also been shown to significantly reduce fasting glucose, improve insulin secretion and sensitivity, and protect pancreatic β cells from oxidative damage [44,47]. Additionally, in obese patients IPA has been negatively correlated with BMI, hs-CRP, hs-IL-6, and it has been reported that could inhibit the accumulation of TG and lower blood lipid levels, markers of progression in HIV infection, this suggest that in future research it will be that in future research it could be promising to look for these associations in PLWHIV, especially in those patients with dyslipidemia and who experiencing weight gain after using ART, conditions associated with an increased risk of T2D [48,49,50].

It has been found to positively modulate GLP-1 secretion, further strengthening its hypoglycemic effect [51]. A prospective study in Finland showed that elevated levels of IPA were associated with a lower risk of developing T2D [44].

Among intestinal bacteria capable of synthesizing IPA are *Peptostreptococcus russellii*, *Lactobacillus* spp., *Clostridium sporogenes*, *Clostridium botulinum*, *Lechevalieria aerocolonigenes*, and *Clostridium paraputrificum* [52,53]. These species may represent therapeutic targets in microbiota modulation strategies to prevent or treat metabolic alterations.

### 3.2. Serotonin and γ-Aminobutyric Acid

Serotonin (5-HT, 5-hydroxytryptamine) is a key neurotransmitter synthesized from tryptophan. Its production begins with the conversion of tryptophan into 5-hydroxytryptophan by the enzyme tryptophan hydroxylase 1, and subsequently into serotonin by aromatic amino acid decarboxylase. Although a fraction is synthesized in the central nervous system, about 90% is generated in the intestine, particularly in the enterochromaffin cells of the colon [25,54]. The GM actively regulates this synthesis. It has been shown that spore-forming intestinal bacteria can induce serotonin production, as evidenced by studies in germ-free mice, which show reduced serotonin levels in serum, colon, and feces compared to mice with conventional microbiota [54]. Among the bacteria described as serotonin producers are: *Streptococcus*, *Escherichia*, *Enterococcus*, *Hafnia alvei*, *Klebsiella pneumoniae*, *Lactobacillus plantarum*, *Morganella morganii*, *Akkermansia*, *Alistipes*, and *Roseburia* [55,56,57] (Figure 1).

In PLWHIV, it has been documented that the imbalance of the GM significantly affects serotonin production. The reduction in symbiotic bacteria that promote its synthesis, together with the diversion of tryptophan catabolism toward the kynurenine pathway, compromises its generation [58,59].

The effects of serotonin in the context of T2D have been contradictory, depending, among other factors, on the receptors it stimulates (5-HT2C, 5-HT1A, SERT) and the organs involved. A study by Al-Zoairy et al. in rat L6 skeletal muscle cells demonstrated that serotonin increases the translocation of glucose transporter type 4 (GLUT4), improving glucose uptake and glycogenesis. This is due to the serotonylation of the small GTPase Rab4, whose activity is stimulated by both insulin and serotonin [60]. On the other hand, at the pancreatic level, this neurotransmitter stimulates insulin secretion through serotonylation of GTPases such as Rab3a and Rab27a and inhibits glucagon release from α-cells [54,61,62]. Low serotonin is often associated with comorbid depression and poorer glycemic control [61,63].

Hyperactivity of the HPA axis, which can be due to oxidative stress, increases glucocorticoid secretion, which in turn inhibits 5-HT synthesis; also, cytokines decrease serotonin synthesis, increasing its consumption and reducing its availability. Experimental models of glucose intolerance in response to a high-fat diet have shown that treatment with 5-HT improves insulin secretion. In PLWHIV, it has been reported that low levels of 5-HT, generally associated with depression in these people, are present oxidative stress and inflammation, and intestinal dysbiosis events that are associated with variations in serotonin, serotonergic dysfunction, and increased intestinal permeability, which may contribute to cardiometabolic risk [59,64]. Undoubtedly, research on 5-HT levels and their association with markers of inflammation and T2D is necessary in the search to advance knowledge of the pathophysiology of this metabolic condition in PLWHIV.

However, other findings indicate that plasma serotonin levels are higher in people with T2D, and that its excess could induce IR and hyperglycemia. In this context, it has been suggested that its inhibition favors thermogenesis in brown adipose tissue and improves the metabolic profile [54,65]. These negative effects of serotonin are based on its action on specific receptors in white adipose tissue, where, through phosphorylation cascades, it increases the activity of hormone-sensitive lipase, enhancing lipolysis and the concentration of free fatty acids in peripheral blood. In addition, it has been demonstrated through murine models that this neurotransmitter induces lipogenesis in visceral white adipose tissue [66].

Moreover, it has been demonstrated in murine models that serotonin can induce hyperglycemia through the release of adrenaline from the adrenal glands, and reduce or increase glycogen synthesis depending on its concentration [67]. Given the evidence of the opposing effects of serotonin, much remains to be elucidated about its role in HIV and the development of metabolic disorders, especially T2D.

**Figure 2:**
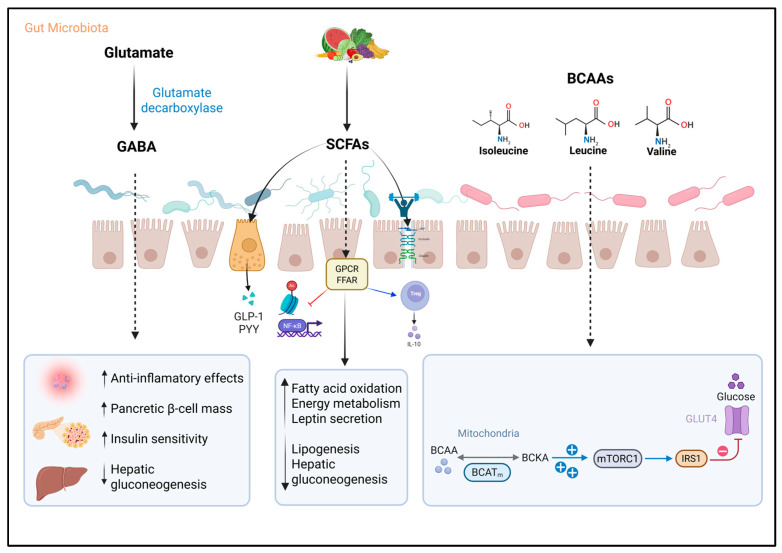
Microbial metabolites and their role in the development of type 2 diabetes in people living with HIV. A schematic representation of various microbial metabolites derived from the gut microbiota, their translocation across the intestinal epithelial barrier, and their immunological and metabolic effects in peripheral tissues. These metabolites include γ-aminobutyric acid (GABA), short-chain fatty acids (SCFAs), and branched-chain amino acids (BCAAs). Branched-chain aminotransferase, mitochondrial (BCATm); branched-chain keto acids (BCKA); free fatty acid receptor (FFAR); glucose transporter type 4 (GLUT4); glucagon-like peptide-1 (GLP-1); G protein-coupled receptors (GPCRs); insulin receptor substrate 1 (IRS1); mechanistic target of rapamycin complex 1 (mTORC1); peptide YY (PYY). Created with BioRender.com (accessed on 12 September 2025).

Gamma-aminobutyric acid (GABA) is an inhibitory neurotransmitter widely produced by intestinal bacteria. The most studied pathway is the one using the enzyme glutamate decarboxylase, encoded by the *gadA* or *gadB* genes, which transforms glutamate into GABA [68,69,70]. Various bacteria possess this capacity, such as *Escherichia coli*, *Listeria monocytogenes*, *Bifidobacterium* spp., *Bacteroides* spp., and lactic acid bacteria like *Lactobacillus*, *Lactococcus*, and *Streptococcus* [70,71,72,73]. HIV infection has been associated with a reduction in GABA-producing intestinal bacteria, such as *Bacteroides* spp. [74] (Figure 2).

GABA acts on specific type A receptors in pancreatic β, α, and δ cells, modulating glucose-induced insulin secretion. Its ability to promote β-cell proliferation and their conversion from α-cells has been demonstrated through inhibition of the Arx factor, favoring the restoration of functional pancreatic mass [75,76]. Additionally, GABA activates the PI3K/AKT pathway (phosphoinositide 3-kinase/protein kinase B), enhancing the expression of insulin receptor substrate 1 (IRS1) and GLUT4, thereby boosting insulin sensitivity and reducing homeostatic model assessment for insulin resistance (HOMA-IR) [76,77]. In the liver, it downregulates genes involved in gluconeogenesis and lipogenesis, reducing hepatic glucose production and lipid accumulation [76,78]. In the immunological realm, it exerts anti-inflammatory effects by reducing the production of cytokines such as TNF-α, IFN-γ, and IL-13, and by limiting helper T cell proliferation, contributing to the resolution of chronic inflammatory processes [76,79]. This highlights the central role of GM in regulating metabolites such as serotonin and GABA, whose alterations are closely linked to both HIV infection and T2D; the association of GABA, GM, and inflammation is an unexplored area in PLWHIV.

### 3.3. Short-Chain Fatty Acids

Short-chain fatty acids (SCFAs), mainly acetate, propionate, and butyrate, are metabolites generated by bacterial fermentation of non-digestible dietary fiber in the colon. Their production largely depends on anaerobic bacteria belonging to the Bacillota and Bacteroidota phyla, such as *Faecalibacterium*, *Roseburia*, *Coprococcus*, and *Eubacterium* [28,29,36]. These compounds not only represent an important energy source for intestinal epithelial cells but also participate in maintaining intestinal barrier integrity, modulating inflammation, and contributing to various host metabolic functions [29,36] (Figure 2).

SCFAs intervene in lipid and carbohydrate metabolism through the activation of G protein-coupled receptors (GPCRs), such as free fatty acid receptor 2 (FFAR2), FFAR3, and GPR109A, which modulate intracellular pathways including extracellular signal-regulated kinases 1 and 2 (ERK1/2) activation, cyclic adenosine monophosphate (cAMP) control, and G protein regulation [36,80]. At the intestinal level, SCFAs have been shown to strengthen the epithelial barrier and increase mucin secretion by goblet cells, as well as intestinal hormones GLP-1 and peptide YY (PYY), involved in appetite control and improved insulin sensitivity [36,80]. Particularly, propionate has been described as protective of the colonic epithelium due to its inhibitory action on class 2 histone deacetylases (HDAC2), thus reducing apoptotic processes in intestinal epithelial cells triggered by oxidative stress at this level [81].

In the immune system, butyrate has shown anti-inflammatory effects through inhibition of the transcription factor NF-κB and induction of IL-10 production [82]. Likewise, butyrate, together with propionate, has inhibitory effects on HDACs, modulating the secretion of incretins (GLP-1, PYY), inflammatory pathways (NF-κB), and metabolic pathways (AMPK), enhancing lymphocyte phenotyping toward regulatory phenotypes. Acetate, the most abundantly produced SCFA, can trigger apoptosis in neutrophils and eosinophils, considerably reducing inflammation both at the intestinal and systemic levels [81,83].

At the molecular level, in adipose tissue, SCFAs have been shown to activate AMP-activated protein kinase (AMPK), induce expression of peroxisome proliferator-activated receptor gamma coactivator 1-alpha (PGC-1α), and stimulate peroxisome proliferator-activated receptor (PPAR) activity, thereby promoting fatty acid oxidation [36,84,85]. They also modulate key metabolic signals such as adipose triglyceride lipase (ATGL) and uncoupling proteins (UCPs), essential in regulating energy metabolism [36,85].

The pleiotropic effect of SCFAs is also evident in many other organs and systems. For example, in the central nervous system, they strengthen the blood–brain barrier and act on hypothalamic receptors, negatively modulating food intake. In skeletal muscle, they increase glucose uptake, lipid oxidation, and improve insulin sensitivity. In the liver, propionate can reduce gluconeogenesis, while acetate and butyrate have been shown to inhibit lipogenesis and stimulate leptin secretion, which could contribute to better regulation of appetite and energy metabolism in T2D patients [86,87,88]. In the pancreas, Hu et al. demonstrated that butyrate prevents the reduction in β-cell mass described in T2D patients, as it inhibits the endoplasmic reticulum stress pathway and thus cellular apoptosis. Findings referring to the positive relationship between SCFA levels and insulin secretion are also consistent [89].

In PLWHIV, whether on ART or ART-naive, a significant decrease in butyrate-producing bacteria such as *Faecalibacterium prausnitzii*, *Roseburia intestinalis*, *Lachnospira*, and others of the Bacillota phylum has been observed, resulting in lower SCFA production [28,29,90,91,92]. This decrease may compromise intestinal barrier function and contribute to microbial translocation and chronic immune activation processes characteristic of the infection. In particular, *Roseburia intestinalis* has shown an inverse correlation with levels of microbial translocation and immune activation markers, suggesting a relevant protective role [93,94].

In T2D patients, a reduction in the abundance of SCFA-producing bacteria, including *Bacteroides*, *Bifidobacterium*, *Faecalibacterium*, *Prevotella*, and *Akkermansia*, has also been reported, associated with lower fecal concentrations of acetate, propionate, and butyrate. This decrease has been linked to increased intestinal permeability, systemic inflammation, and insulin resistance, conditions that favor hyperglycemia, increased appetite, and altered lipid metabolism [86].

On the other hand, recent research has demonstrated that these compounds have a dual effect, especially in PLWHIV, sometimes favoring the development of T2D. The pan-inhibitory effect of SCFA HDACs, particularly butyrate, could facilitate latent virus reactivation by inducing epigenetic changes in chromatin that favor proviral DNA transcription [95]. Additionally, this compound has been described as having pro-apoptotic effects and the ability to persistently activate the NLRP3 inflammasome, which contributes to disruption of the intestinal barrier, especially in the presence of a dysbiotic environment dominated by Pseudomonadota phylum bacteria, characteristic of PLWHIV [96].

### 3.4. Branched-Chain Amino Acids

Branched-chain amino acids (BCAAs), which include leucine, isoleucine, and valine, are distinguished by their branched aliphatic side chains and are part of the essential amino acids that can also be synthesized, in part, by the GM. The latter is the main source of circulating BCAAs, as it regulates their concentrations through biosynthesis and modification of absorption. In recent years, these compounds have been recognized as important biomarkers of IR, as well as predictors of the risk of developing T2D and cardiovascular diseases [97,98]. Several clinical studies have shown a correlation between elevated plasma BCAA concentrations and decreased insulin sensitivity, which increases the likelihood of progression to T2D [98,99]. For example, a systematic review including meta-analysis conducted by Ramzan et al. observed a statistically significant positive association between the levels of these three amino acids and the development of T2D, as well as a positive temporal association between these elevated levels and the risk of developing T2D [100] (Figure 2).

Among the pathophysiological pathways that could explain the relationship between BCAAs and the development of T2D, it is proposed that in states of IR, there is a deficient inhibition of proteolysis, which contributes to the increase in circulating BCAAs together with those derived from the GM. Likewise, altered signaling of adiponectin, a hormone involved in energy metabolism regulation, is associated with lower degradation of these amino acids in peripheral tissues. Under these conditions, incomplete catabolism of BCAAs occurs, leading to the accumulation of intermediate metabolites such as α-ketoisocaproic acid. This compound can interfere with insulin signaling, promote a proinflammatory environment, and inhibit insulin-induced glucose transport in myocytes, an effect mediated by the mitochondrial enzyme branched-chain aminotransferase 2 (BCAT2) [101,102].

Regarding molecular mechanisms, BCAAs, especially leucine, and to a lesser extent isoleucine, can continuously activate a cellular pathway known as mechanistic target of rapamycin complex 1 (mTORC1). When this pathway remains active for too long, it negatively affects the function of a key protein in insulin signaling called IRS1. This occurs because mTORC1 causes IRS1 to be phosphorylated at specific sites, which blocks its normal function. As a result, insulin cannot perform its function, which decreases glucose uptake by cells and reduces glycogen production, thus contributing to IR [103].

Particularly, isoleucine is associated with increased IR, especially in skeletal muscle tissue, as it has been linked to increased muscle mass associated with lipid accumulation in this tissue. In the case of valine, its catabolic intermediate, 3-hydroxyisobutyric acid (3-HIB), promotes the uptake and accumulation of fatty acids in muscle and adipocytes, leading to lipotoxicity and deterioration of insulin signaling, an effect observed in both animal models and humans with overweight, obesity, or T2D [104].

*Prevotella copri* and *Bacteroides vulgatus* are gut bacteria with a high capacity to synthesize BCAAs, as well as *Clostridium sporogenes*. The elevated presence of these bacteria in the intestine has been associated with higher plasma BCAA levels and an increase in IR [105,106]. On the other hand, *Faecalibacterium prausnitzii*, *Ruminococcus gnavus*, *Eubacterium siraeum*, and *Butyrivibrio crossotus* have been implicated in increased absorption of these amino acids; that is, the greater their abundance in the GM, the lower their concentrations in blood and the lower the IR [106].

Few studies specifically address the role of BCAAs in PLWHIV and their relationship with metabolic alterations. However, the available evidence, although limited, demonstrates a solid and relevant association between the levels of these metabolites, intestinal dysbiosis, and IR, suggesting a significant impact on metabolic pathophysiology and chronic inflammation in the context of HIV. For example, a recent study showed that the altered microbiota in PLWHIV modulates BCAA concentration, linking it with inflammatory and metabolic processes that contribute to the development of T2D in this population group [24].

### 3.5. Bile Acids

**Figure 3:**
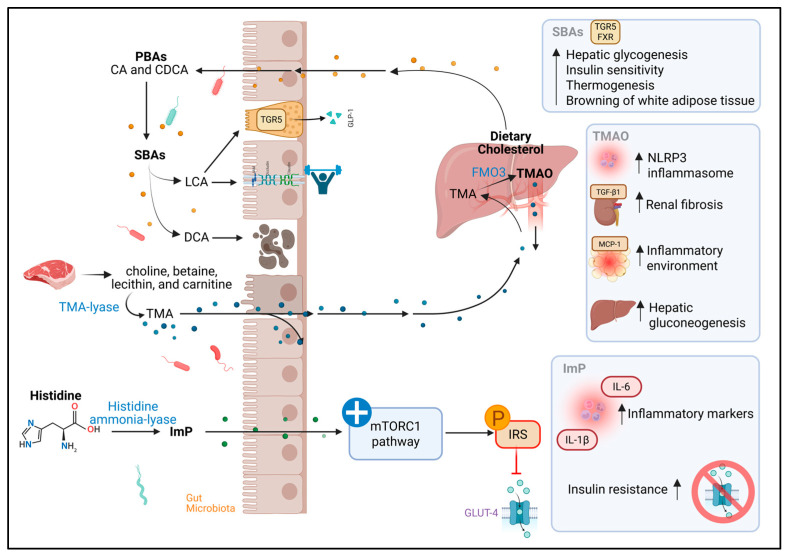
The role of microbial metabolites such as secondary bile acids (SBAs), imidazole propionate (ImP), and trimethylamine N-oxide (TMAO) in the development of type 2 diabetes in people living with HIV. Primary bile acids (PBAs); cholic acid (CA); chenodeoxycholic acid (CDCA); deoxycholic acid (DCA); lithocholic acid (LCA); flavin-containing monooxygenase 3 enzyme (FMO3); monocyte chemoattractant protein-1 (MCP-1); NOD-like receptor family pyrin domain containing 3 (NLRP3); transforming growth factor beta 1 (TGF-β1); trimethylamine (TMA); Takeda G protein-coupled receptor 5 (TGR5); farnesoid X receptor (FXR). ↑ increase; ↓ decrease. Created with BioRender.com (accessed on 12 September 2025).

Primary bile acids (BAs), such as cholic acid (CA) and chenodeoxycholic acid (CDCA), are synthesized in the liver from cholesterol. Once synthesized, they are transported to the intestine, where they come into contact with the GM, which converts them into more hydrophobic secondary bile acids, such as deoxycholic acid (DCA) and lithocholic acid (LCA), through reactions such as deconjugation and 7α-dehydroxylation [36,107,108]. Various bacteria are involved in this process. For example, *Bacteroides* and *Enterococcus* participate in the initial deconjugation, whereas bacteria such as *Lactobacillus*, *Bifidobacterium*, *Staphylococcus*, *Clostridium perfringens*, and members of the Lachnospiraceae and Ruminococcaceae families are involved in the conversion to secondary acids using enzymes such as bile salt hydrolase and 7α-dehydroxylase [107,108] (Figure 3).

Although they have traditionally been attributed to digestive functions, emulsifying fats and facilitating the absorption of fat-soluble vitamins, it is now recognized that BAs also act as signaling molecules. They activate receptors such as the farnesoid X receptor (FXR) and Takeda G protein-coupled receptor 5 (TGR5), which trigger a series of metabolic effects [108,109]. For example, TGR5 activation by secondary BAs stimulates GLP-1 release from enteroendocrine cells, promoting insulin secretion, delaying gastric emptying, and reducing appetite. FXR, in turn, promotes hepatic glycogen synthesis and improves insulin sensitivity. Moreover, both receptors contribute to thermogenesis and the browning of white adipose tissue [108,109].

BAs also modulate intestinal barrier integrity. While DCA, at high concentrations, can be cytotoxic, alter Zonula Occludens-1 (ZO-1) protein, and increase intestinal permeability, LCA appears to exert a protective effect, counteracting inflammatory damage induced by TNF-α [109,110,111].

Scientific literature on BA profiles in PLWHIV is still scarce. However, one study revealed elevated concentrations of both primary and secondary BAs in PLWHIV who also had chronic hepatitis C virus coinfection and a history of depression. This finding was related to significant alterations in GM composition, suggesting a possible dysfunction in the bacterial metabolism of BAs in this patient group [112]. Although the functional consequences of this dysbiosis on the microbiota–bile acid axis have not been precisely determined, these results point to a relevant area of research, given the immunomodulatory and metabolic role of BAs.

In the context of T2D, BA profiles show alterations that could have important pathophysiological implications. One consequence of intestinal dysbiosis in T2D is a lower conversion of primary to secondary BAs, leading to their accumulation. For example, an increase in CDCA has been observed to reduce very low-density lipoprotein production and lower plasma triglyceride levels, effects potentially beneficial for the lipid profile [113]. However, studies in individuals with IR have shown a trend toward higher total BA concentrations, although the results remain heterogeneous [114,115]. These findings have sparked interest in BAs as potential therapeutic targets for T2D, both for their ability to activate key metabolic receptors (FXR and TGR5) and for their effects on inflammation, intestinal integrity, and energy homeostasis [115].

### 3.6. Trimethylamine N-Oxide

Trimethylamine (TMA) is a compound generated exclusively by the GM from the metabolism of dietary nutrients such as choline, betaine, lecithin, and carnitine, which are especially abundant in animal-derived foods like red meat. This transformation depends on bacteria that express the enzyme TMA-lyase, among which species, such as *Anaerococcus hydrogenalis*, *Clostridium asparagiforme*, *Clostridium hathewayi*, *Clostridium sporogenes*, *Escherichia fergusonii*, *Proteus penneri*, *Providencia rettgeri*, and *Salmonella enterica*, have been identified. The conversion of carnitine to TMA, on the other hand, has been mainly associated with bacteria of the phylum Pseudomonadota and particularly from the family Prevotellaceae [116,117] (Figure 3).

Once produced, TMA travels to the liver, where it is oxidized by flavin-containing monooxygenase 3 (FMO3), becoming trimethylamine N-oxide (TMAO), a metabolite that has gained relevance due to its involvement in metabolic processes and chronic diseases [118,119,120]. It has been described that TMAO levels may vary depending on GM composition, being higher when Bacillota predominate over Bacteroidota [28,29]. They have also been linked to a greater abundance of genera such as *Mitsuokella*, *Fusobacterium*, *Desulfovibrio*, and the archaeon *Methanobrevibacter smithii* [121].

At the cellular level, TMAO activates the PKR-like endoplasmic reticulum kinase (PERK) signaling pathway, promoting hyperglycemia and IR. In adipose tissue, it fosters an inflammatory environment by increasing the expression of monocyte chemoattractant protein-1 (MCP-1) and decreasing IL-10 levels. In the liver, it stimulates gluconeogenesis, reduces glucose uptake, and depletes glycogen stores, negatively impacting glucose homeostasis [122]. It has also been implicated in renal fibrosis processes by inducing the expression of transforming growth factor beta 1 (TGF-β1) and alpha-smooth muscle actin (α-SMA), as well as in the activation of the NOD-like receptor family pyrin domain containing 3 (NLRP3) inflammasome, enhancing the release of inflammatory cytokines such as IL-1β and IL-18 [123]. In addition to these actions, TMAO can alter cellular pathways such as calcium signaling, cholesterol metabolism, and bile acid synthesis, contributing to foam cell formation and the development of atherosclerosis [28,124].

In PLWHIV, findings on circulating TMAO levels have been variable. Some studies have reported a higher proportion of TMAO in relation to ART use, suggesting a possible impact of these drugs on its metabolism. Nevertheless, other studies have not found significant differences in this metabolite’s levels between individuals with and without HIV, nor clear correlations with parameters such as CD4^+^ T cell count or ART use [125,126,127]. It is worth noting that most available studies do not delve into the bacterial composition responsible for TMA production in this population, which limits the understanding of how the specific GM of PLWHIV might be modulating TMAO levels. Even so, recent research has begun to explore the possible relationship between this metabolite and HIV-specific features, such as chronic inflammatory state or dysbiosis, and its eventual link with associated diseases, including T2D [24,28,125].

In patients with T2D, TMAO has been widely studied for its potential role in the development and progression of the disease. Elevated levels of this metabolite have been associated with IR, impaired glucose tolerance, and greater systemic inflammation. Furthermore, some studies have reported that in patients with T2D, high TMAO concentrations may be linked to increased risk of mild cognitive impairment and a higher incidence of cardiovascular events [119,128].

### 3.7. Imidazole Propionate

Imidazole propionate (ImP) is a metabolite derived from the bacterial degradation of histidine, and its formation pathway is highly conserved among different species of gut bacteria, underscoring its evolutionary relevance. This pathway begins with the action of the enzyme histidine ammonia-lyase, which removes an ammonium group from histidine to generate urocanate. Subsequently, urocanate hydratase converts this intermediate into ImP through a hydration reaction [129] (Figure 3).

From a functional perspective, it has been demonstrated that ImP can interfere with cellular metabolism. Koh et al. documented that this metabolite activates the mitogen-activated protein kinase (MAPK) p38γ, which triggers phosphorylation of the p62 protein, abnormally activating the mTORC1 pathway, as evidenced by phosphorylation at serine 2448. This activates ribosomal protein S6 kinase beta-1 (S6K1), which induces defective phosphorylation of IRS, promoting its degradation and thus favoring the development of IR [130].

In terms of microbiota, the production of ImP has been linked to certain bacterial species such as *Clostridium baumannii*, *Clostridium parasymbiotics*, *Ruminococcus gnavus*, and *Veillonella*, many of which have been associated with states of intestinal dysbiosis. In parallel, its accumulation has shown positive correlations with elevated levels of systemic inflammatory markers, suggesting a potentially detrimental role in the host’s immunometabolic balance [130,131].

In PLWHIV, ImP has gained attention as a possible indicator of dysbiosis and cardiovascular risk. Recent studies have identified that *Ruminococcus gnavus* and *Veillonella*, ImP-producing bacteria, are increased in this population group [29,131]. Within the framework of the COCOMO study, these microbial species were associated with obstructive coronary disease in PLWHIV, leading to the proposal of ImP as a potential circulating biomarker for this condition [132]. A complementary study in women with HIV found that ImP levels were associated with carotid atherosclerosis and an altered GM. This research also identified bacterial species not previously related to the production of this metabolite, as well as the presence of the *hutH* gene, which encodes histidine ammonia-lyase, a key enzyme in its biosynthetic pathway [133]. Based on these findings, a microbial score was developed based on the abundance of species linked to ImP, which showed positive correlations with arterial plaques and inflammatory biomarkers, reinforcing the hypothesis that this metabolite may reflect both dysbiosis and cardiovascular risk in PLWHIV, regardless of sex or mode of transmission [29,133].

In the context of T2D, ImP has been associated with key mechanisms involved in altered insulin signaling and energy metabolism [130]. Additionally, a multicenter cohort study reported higher levels of ImP in individuals with prediabetes and T2D, especially in those with a microbial profile dominated by the Bacteroides 2 enterotype and low microbial genetic diversity. This microbial pattern has been linked to a dysbiotic intestinal ecosystem, poor in anti-inflammatory species, and with a higher presence of potentially pathogenic bacteria. The positive correlation between ImP levels and systemic inflammatory markers in these individuals suggests that this metabolite acts not only as a metabolic mediator but also as a modulator of the chronic inflammatory state characteristic of T2D [130,131]

It is evident that most reports in the scientific literature on functional microbial metabolites and their relationship with T2D have focused on the general population. Despite this, a considerable number of recent investigations have described altered metabolomic profiles in PLWHIV, a population highly vulnerable to developing metabolic diseases. These studies have concluded that these metabolites play a significant role as the link between documented intestinal dysbiosis and various markers of systemic inflammation, immunometabolic dysfunction, and IR.

In general, the metabolomic profile of PLWHIV includes preferential activation of the kynurenine pathway at the expense of indole production, imbalances in the production of neurotransmitters such as GABA and serotonin, reductions in SCFA, elevated levels of BCAA, and increased concentrations of secondary bile acids, TMAO, and ImP.

Despite the relevance of this topic, there are few studies that simultaneously integrate the analysis of microbiota, metabolites, and various markers of glucose homeostasis in PLWHIV. The findings reported so far consistently reinforce the link between intestinal dysbiosis, an altered metabolomic profile, and low-grade systemic inflammation, playing a relevant role in the progression toward prediabetic or diabetic states. This knowledge underscores the need and opens pathways for studies that address these axes more deeply and integrally within the complex clinical context characterized by HIV infection.

### 3.8. Therapeutic and Diagnostic Opportunities

Recent evidence highlights the role of GM-derived metabolites as promising biomarkers of IR and particularly T2D, especially in PLWHIV. For instance, increased kynurenine/tryptophan ratios (an indicator of activation of the inflammatory kynurenine pathway), BCAAs (isoleucine, leucine, and valine), TMAO, and ImP have been identified in individuals living with both T2D and HIV, being associated with the incidence of this metabolic disease [23,24]. This underscores the potential of profiling microbial metabolites as a non-invasive tool for early stratification and monitoring of metabolic alterations, particularly in populations where disease progression cannot be explained by traditional risk factors described in the literature.

In the case of IPA, an experimental model demonstrated that a diet rich in IPA improved glucose metabolism and significantly reduced HOMA-IR, fasting glucose, insulin, and IR, effects that may be achieved through its efficacy in regulating the secretion of (GLP)-1 cells and thus increasing B cell regeneration and proliferation. On the other hand, IPA intervention could reduce the inflammatory event associated with HIV infection and T2D [51,134,135,136].

Regarding therapeutics, strategies aimed at modulating GM composition have attracted special interest due to their beneficial outcomes in various diseases. Supplementation with personalized probiotics, implementation of prebiotic-rich diets, and fecal microbiota transplantation (FMT) have enabled the adequate restoration of both the microbial GM profile and systemic metabolites, representing important therapeutic targets in metabolic diseases [36]. Similarly, innovative studies have explored probiotics capable of inducing differential production of beneficial metabolites such as SCFAs, which strengthen the intestinal epithelial barrier, exert anti-inflammatory effects, and contribute to proper glucose homeostasis [137]. Moreover, supplementation with certain metabolites, such as butyrate, has promoted restoration of microbial diversity and contributed to improved insulin sensitivity [138].

In the POUNDS Lost trial, researchers included overweight and obese adults assigned to hypocaloric diets with different macronutrient compositions. Participants whose diets most significantly reduced choline and L-carnitine intake were associated with significant improvements in IR indicators, such as the HOMA-IR [139].

It is important to emphasize that evidence in PLWHIV remains considerably limited, further highlighting the importance of initial experimental studies that consider therapeutic strategies integrating the approaches as adjuncts to ART, thereby mitigating their adverse metabolic effects. In the long term, integrating metabolomics with clinical and microbiota data could serve as a starting point for developing precision medicine strategies in PLWHIV; that is, characterization of individual metabolomic and intestinal microbial signatures would allow for prediction and monitoring of diverse metabolic alterations while identifying therapeutic targets. To achieve this, longitudinal studies with designs capable of establishing causality and standardized methodologies for using metabolomics as biomarkers are required. This is a rapidly evolving field that offers opportunities to bridge molecular findings with clinical applications.

## 4. Conclusions

The evidence highlights the complex interplay between the immune response, GM, and the various microbial metabolites derived from it, constituting a key axis in the pathophysiology and the development of T2D beyond the classic metabolic risk factors, especially in a highly vulnerable population such as PLWHIV. These metabolites have the capacity to variably influence immunometabolic balance, depending on their nature and the molecular mechanisms through which they act in peripheral tissues. From those resulting from tryptophan catabolism to compounds such as TMAO, GABA, or SCFAs, each represents a complex interaction between the state of dysbiosis, chronic low-grade inflammation, and the various metabolic alterations associated with the infection. In this context, it is essential to consider the potential of GM and its metabolites not only as biomarkers but also as elements amenable to early intervention to prevent the progression of glycemic dysregulation. However, current studies present limitations in design, highlighting the need for longitudinal research to establish causal relationships. Recognizing these interactions could guide comprehensive, innovative, and personalized therapeutic strategies that not only modulate glycemia but also promote the restoration of microbial balance and immunity, offering a comprehensive approach to improving metabolic health in this vulnerable population.

## Figures and Tables

**Table 1:** Gut microbiota-derived metabolites: sources and relevance in HIV and type 2 diabetes.

Metabolites	Main Microbial Sources	Key Metabolic Pathways	Relevance in HIV	Relevance in T2D
Kynurenine pathway-derived metabolites	*Pseudomonas*, *Xanthomonas*, *Burkholderia*, *Stenotrophomonas*, and *Shewanella*, and members of the genus *Bacillus*	Multiple pathways promoting oxidative stress, chronic immune activation, and inflammation	Associated with disease progression, CD4^+^ T cell depletion, and systemic immune dysfunction. An imbalance between Th17 and Treg cells exacerbates immunosuppression and intestinal epithelial damage	Linked to increased IR through chronic low-grade inflammation, oxidative stress, and decreased tryptophan availability for the synthesis of protective metabolites
Indole Pathway-derived metabolites	*Roseburia*, *Eubacterium*, *Lachnospira*, *Coprobacter Peptostreptococcus russellii*, *Lactobacillus* spp., *Clostridium sporogenes*, and *Clostridium paraputrificum*	Acts on AhR and PXR receptors, modulating immune functions, intestinal barrier integrity, and cytokine production	Reduced production is associated with decreased IL-12, impaired intestinal epithelial barrier integrity, and dysbiosis	Protective metabolite associated with lower fasting glucose, enhanced insulin secretion, and improved insulin sensitivity. Levels are reduced in T2D
Serotonin	*Streptococcus*, *Escherichia*, *Enterococcus*, *Hafnia alvei*, *Klebsiella pneumoniae*, *Lactobacillus plantarum*, *Morganella morganii*, *Akkermansia*, *Alistipes*, and *Roseburia*	- Serotonylation of specific GTPases enhances GLUT4 translocation, increases insulin secretion, and inhibits glucagon release	An imbalance of GM significantly affects serotonin production	Results are contradictory
		- Can induce hyperglycemia through adrenaline release and alter glycogen synthesis		
GABA	*Escherichia coli*, *Listeria monocytogenes*, *Bifidobacterium* spp., *Bacteroides* spp., and lactic acid bacteria like *Lactobacillus*, *Lactococcus*, and *Streptococcus*	Acts on specific type A receptors, modulating glucose-induced insulin secretion. Activates the PI3K/AKT pathway, enhancing IRS1 and GLUT4 expression	Exhibits anti-inflammatory effects. HIV is associated with reduced abundance of GABA-producing intestinal bacteria	Increase insulin sensitivity and secretion. Reduces hepatic glucose production and lipid accumulation
SCFAs	*Faecalibacterium*, *Roseburia*, *Coprococcus*, and *Eubacterium*	Activates GPCRs, inhibits HDAC2, stimulates PPAR activity for fatty acid oxidation, and modulates key metabolic regulators (ATGL, UCPs)	Significant decrease in SCFA-producing bacteria, which may compromise intestinal barrier function and contribute to microbial translocation and chronic immune activation characteristic of the infection	Enhances intestinal barrier and hormone secretion (GLP-1, PYY, leptin), improves insulin sensitivity, glucose uptake, and lipid metabolism, and preserves β-cell mass
BCAAs	*Prevotella copri*, *Bacteroides vulgatus*, and *Clostridium sporogenes*	Incomplete catabolism of BCAAs leads to the accumulation of intermediate metabolites such as α-ketoisocaproic acid. BCAAs can continuously activate the mTORC1 cellular pathway, which negatively affects the function of IRS1	Strong and relevant association with intestinal dysbiosis and IR, suggesting a significant impact on metabolic pathophysiology and chronic inflammation in the context of HIV	Elevated BCAAs are associated with IR, impaired glucose uptake, and lipotoxicity. Accumulation of catabolic intermediates disrupts insulin signaling. Gut bacteria modulate circulating BCAA levels, influencing T2D risk
Bile Acids (Primary and Secondary)	*Lactobacillus*, *Bifidobacterium*, *Staphylococcus*, *Clostridium perfringens* and members of the Lachnospiraceae y Ruminococcaceae	FXR/TGR5 activation: improves glucose metabolism, insulin sensitivity, appetite regulation, and thermogenesis; DCA cytotoxic at high levels; LCA protective	Elevated concentrations of primary and secondary BAs in PLWHIV with chronic hepatitis C virus coinfection and a history of depression, related to significant alterations in intestinal microbiota composition. Much remains to be investigated	Trend toward higher total BA concentrations. Many results are contradictory
TMAO	*Anaerococcus hydrogenalis*, *Clostridium asparagiforme*, *Clostridium hathewayi*, *Clostridium sporogenes*, *Escherichia fergusonii*, *Proteus penneri*, *Providencia rettgeri* and *Salmonella enterica*	Activates PERK pathway; promotes hyperglycemia and IR; increases adipose inflammation (↑ MCP-1, ↓ IL-10); stimulates hepatic gluconeogenesis; implicated in renal fibrosis and NLRP3 inflammasome activation	Many studies have reported higher TMAO levels, especially associated with ART, but this has not been consistent in other research	TMAO has been widely studied for its potential role in the development and progression of disease. Elevated levels of this metabolite have been associated with IR, impaired glucose tolerance, and increased systemic inflammation
ImP	*Clostridium baumannii*, *Clostridium parasymbiotics*, *Ruminococcus gnavus*, and *Veillonella*	Activates MAPK p38γ, which triggers phosphorylation of the p62 protein, abnormally activating the mTORC1 pathway and inducing defective phosphorylation of IRS, promoting its degradation and thus favoring the development of IR	In PLWHIV, ImP has gained attention as a potential indicator of dysbiosis and cardiovascular risk. Several studies report consistent findings linking this metabolite to intestinal dysbiosis in HIV	Positive correlations have been reported between ImP levels, systemic inflammatory markers, and IR in people with T2D

Abbreviations: human immunodeficiency virus (HIV); type 2 diabetes (T2D); insulin resistance (IR); aryl hydrocarbon receptor (AhR); pregnane X receptor (PXR); glucose transporter type 4 (GLUT4); gut microbiota (GM); γ-aminobutyric acid (GABA); phosphoinositide 3-kinase/protein kinase B (PI3K/AKT); short-chain fatty acids (SCFAs); G protein-coupled receptors (GPCRs); histone deacetylase 2 (HDAC2); peroxisome proliferator-activated receptor (PPAR); adipose triglyceride lipase (ATGL); uncoupling proteins (UCPs); glucagon-like peptide-1 (GLP-1); peptide YY (PYY); branched-chain amino acids (BCAAs); mechanistic target of rapamycin complex 1 (mTORC1); insulin receptor substrate 1 (IRS1); farnesoid X receptor (FXR); Takeda G-protein-coupled receptor 5 (TGR5); deoxycholic acid (DCA); lithocholic acid (LCA); bile acids (BAs); people living with HIV (PLWHIV); PKR-like endoplasmic reticulum kinase (PERK); monocyte chemoattractant protein-1 (MCP-1); NOD-like receptor family pyrin domain containing 3 (NLRP3); trimethylamine N-oxide (TMAO); antiretroviral therapy (ART); mitogen-activated protein kinase (MAPK); imidazole propionate (ImP). Data are compiled from the current literature on gut microbiota-derived metabolites in HIV and T2D. ↑ increase; ↓ decrease.

## Data Availability

Data sharing is not applicable to this article as no new data were created or analyzed in this study.

## Abbreviations

The following abbreviations are used in this manuscript:

HIV	Human immunodeficiency virus
AIDS	Acquired immunodeficiency syndrome
PLWHIV	People living with HIV
ART	Antiretroviral therapy
T2D	Type 2 diabetes
GM	Gut microbiota
GALT	Gut-associated lymphoid tissue
LPS	Lipopolysaccharides
Trp-KYN	Kynurenine pathway
TDO	Tryptophan 2,3-dioxygenase
IDO	Indoleamine 2,3-dioxygenase
KYN	Kynurenine
KYNA	Kynurenic acid
AA	Anthranilic acid
3-HK	3-hydroxykynurenine
QUIN	Quinolinic acid
TNF-α	Tumor Necrosis Factor alpha
IL-1β	Interleukin 1 beta
IR	Insulin resistance
IAA	Indole-3-acetic acid
IAld	Indole-3-aldehyde
IPA	Indole-3-propionic acid
IAAld	Indole-3-acetaldehyde
AhR	Aryl hydrocarbon receptor
PXR	Pregnane X receptor
TFF3	Trefoil factor family 3
RELMβ	Resistin-like molecule beta
5-HT	5-hydroxytryptamine
HOMA-IR	Homeostatic model assessment of insulin resistance
GLP-1	Glucagon-like peptide-1
GABA	γ-Aminobutyric acid
PI3K/AKT	Phosphoinositide 3-kinase/protein kinase B
IRS1	insulin receptor substrate 1
GLUT4	Glucose transporter type 4
IFN-γ	Interferon gamma
SCFAs	Short-chain fatty acids
GPCRs	G protein-coupled receptors
FFAR	Free fatty acid receptor
ERK1/2	Extracellular signal-regulated kinases 1 and 2
cAMP	Cyclic adenosine monophosphate
PYY	Peptide YY
AMPK	AMP-activated protein kinase
PGC-1α	Peroxisome proliferator-activated receptor gamma coactivator 1-alpha
PPARs	Peroxisome proliferator-activated receptors
ATGL	Adipose triglyceride lipase
UCPs	Uncoupling proteins
PWT2D	People with type 2 diabetes
BCAAs	Branched-chain amino acids
BCAT2	Branched-chain aminotransferase 2
mTORC1	Mechanistic target of rapamycin complex 1
BAs	Bile acids
CA	Cholic acid
CDCA	Chenodeoxycholic acid
DCA	Deoxycholic acid
LCA	Lithocholic acid
TGR5	Takeda G-protein-coupled receptor 5
ZO-1	Zonula Occludens-1
TMA	Trimethylamine
FMO3	Flavin-containing monooxygenase 3 enzyme
TMAO	Trimethylamine N-oxide
PERK	PKR-like Endoplasmic Reticulum Kinase
MCP-1	Monocyte chemoattractant protein-1
TGF-β1	Transforming growth factor beta 1
α-SMA	Alpha-smooth muscle actin
NLRP3	NOD-like receptor family pyrin domain-containing 3
ImP	Imidazole propionate
MAPK	Mitogen-activated protein kinase
S6K1	Ribosomal protein S6 kinase beta-1
